# Perirhinal cortex lesions impair tests of object recognition memory but spare novelty detection

**DOI:** 10.1111/ejn.13106

**Published:** 2015-11-24

**Authors:** Cristian M. Olarte‐Sánchez, Eman Amin, E. Clea Warburton, John P. Aggleton

**Affiliations:** ^1^ Rowett Institute of Nutrition and HealthInstitute of Medical SciencesUniversity of AberdeenForesterhillUK; ^2^ School of PsychologyCardiff University70 Park PlaceCardiffCF10 3ATUK; ^3^ Department of Physiology and PharmacologyMRC Centre for Synaptic PlasticityBristol UniversityBristolUK

**Keywords:** familiarity, habituation, hippocampus, parahippocampal cortex, recognition memory

## Abstract

The present study examined why perirhinal cortex lesions in rats impair the spontaneous ability to select novel objects in preference to familiar objects, when both classes of object are presented simultaneously. The study began by repeating this standard finding, using a test of delayed object recognition memory. As expected, the perirhinal cortex lesions reduced the difference in exploration times for novel vs. familiar stimuli. In contrast, the same rats with perirhinal cortex lesions appeared to perform normally when the preferential exploration of novel vs. familiar objects was tested sequentially, i.e. when each trial consisted of only novel or only familiar objects. In addition, there was no indication that the perirhinal cortex lesions reduced total levels of object exploration for novel objects, as would be predicted if the lesions caused novel stimuli to appear familiar. Together, the results show that, in the absence of perirhinal cortex tissue, rats still receive signals of object novelty, although they may fail to link that information to the appropriate object. Consequently, these rats are impaired in discriminating the source of object novelty signals, leading to deficits on simultaneous choice tests of recognition.

## Introduction

The present study examined why the rat perirhinal cortex is vital for recognition memory, i.e. the ability to detect when a stimulus is repeated. The rationale arose from the finding that perirhinal cortex lesions consistently impair the spontaneous discrimination of a novel object from a familiar object (Ennaceur *et al*., 1996; Dere *et al*., 2007; Winters *et al*., 2008; Warburton & Brown, 2015). Such tests are based on the preferential exploration of novel objects. Prior to any such recognition test, rats must first be familiarized with one of the objects (sample phase), which is then subsequently paired with a novel object in the test phase. It is therefore most surprising that rats with perirhinal cortex lesions often show intact levels of object exploration during the sample phase, i.e. when only novel stimuli are present, despite their subsequent recognition deficit (e.g. Ennaceur *et al*., 1996; Aggleton *et al*., 1997; Moran & Dalrymple‐Alford, 2003; Winters *et al*., 2004; Barker *et al*., 2007; Bartko *et al*., 2007a,b; Mumby *et al*., 2007; Albasser *et al*., 2009, 2015; McTighe *et al*., 2010). Such behaviour during the sample phase suggests normal detection of novelty, but this seemingly spared ability contrasts with the discrimination deficit found in the subsequent spontaneous novelty preference test.

**Figure 1:**
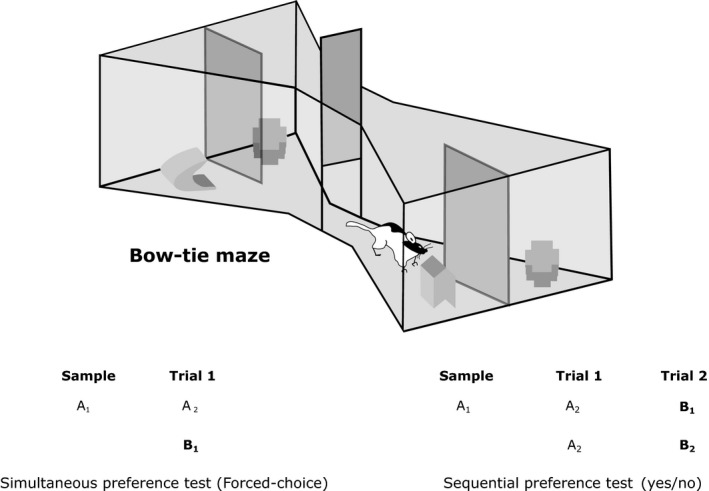
Upper: depiction of the bow‐tie maze with a sliding door separating the two halves and objects covering the food wells. Lower: schematic showing the difference between simultaneous and sequential preference tests of object recognition memory. Different objects are represented by different letters. The numbers indicate the use of duplicate and triplicate objects. For the sequential test, the order of novel and familiar object pairs is counterbalanced. Novel objects are in bold, i.e. when first presented.

One possible explanation for this apparent anomaly is that perirhinal cortex lesions disrupt the ability to distinguish the particular source of novelty signals, but information that a novel object is present remains, so preserving exploration levels. This potential explanation prompted a series of experiments that compared the ability to determine that a specific stimulus is novel with the ability to detect that a novel stimulus is present. To test this possibility, the performance of rats with perirhinal cortex lesions was contrasted in two behavioural procedures, only the first of which requires the rat to discriminate the specific novel stimulus. This first procedure (Experiments 1–4) tested the spontaneous preference for novel objects over familiar objects, when both are presented simultaneously, i.e. ‘forced‐choice’ (Fig. 1) (Ennaceur & Delacour, 1988; Steckler *et al*., 1998; Dere *et al*., 2007; Winters *et al*., 2008). The second procedure (Experiments 4 and 5) involved sequentially presenting pairs of objects that were either both novel or both familiar, i.e. a ‘yes/no’ choice (Fig. 1). Here, recognition memory is reflected in higher exploration levels for novel objects than for familiar objects (McTighe *et al*., 2010). Whereas some sequential tests have found perirhinal cortex lesion deficits (McTighe *et al*., 2010; Romberg *et al*., 2012), others have not (Albasser *et al*., 2011a, 2015).

**Figure 2:**
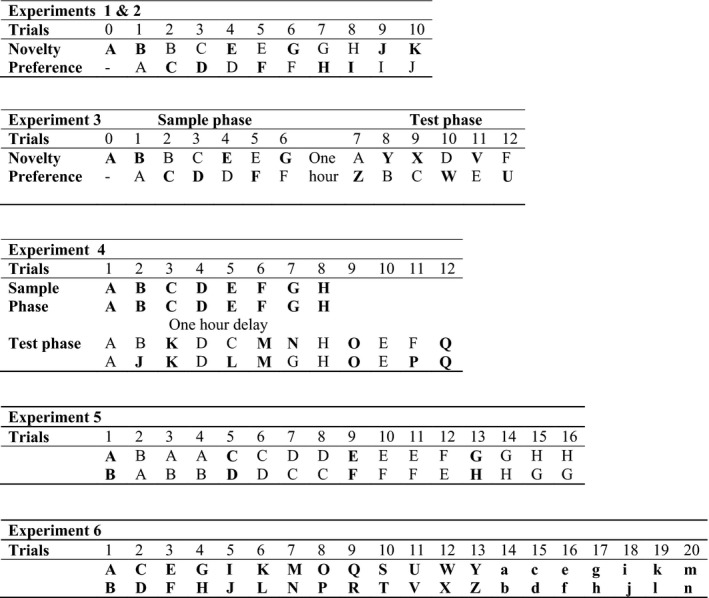
Schematic diagram showing the sequences of object presentations in Experiments 1–6. Different objects are represented by different letters and by changes in case (Experiment 6 only). Novel objects are in bold, i.e. when first presented. Experiments 3 and 4 involved a separate sample phase 1‐h prior to the test phase. All other experiments were integrated in a single phase. Within an experiment, duplicates of the same object were used. Different objects were used in the separate experiments, despite the repeated use of the same letters in this figure.

In the present study, we therefore devised a behavioural protocol that incorporated both simultaneous and sequential procedures within the same session (Experiment 4). Consequently, the test phase compared three types of object pairing: novel with familiar (‘simultaneous’), novel with novel (‘sequential’), and familiar with familiar (‘sequential’) (Fig. 2). In this way, the outcomes from both procedures could be compared directly. Additional experiments then contrasted levels of exploration when rats were given only novel or only familiar stimuli to explore.

## Materials and methods

Two groups of rats with perirhinal cortex lesions (cohort A and cohort B) and their controls were studied. Although cohort A was used in the majority of experiments, the second cohort was used for a final study on the exploration levels for novel objects.

### Animals

Adult male Lister hooded rats (*Rattus norvegicus*) supplied by Harlan Olac (Bicester, UK) were used in all experiments. The rats were housed in pairs, and water was provided *ad libitum* throughout the study. All experiments were performed in accordance with the UK Animals (Scientific Procedures) Act (1986) and associated guidelines, as well as EU directive 2010/63/EU. The study was also approved by local ethical review committees at Cardiff University. Both cohorts were kept on a 12‐h : 12‐h light/dark cycle. Cardboard tubes were present in the home cages for enrichment. Cohort A comprised 29 rats, weighing between 290 g and 350 g at the time of surgery. Cohort B comprised 31 adult male rats, weighing between 290 g and 340 g at the time of surgery.

### Surgical procedures

Cohort A consisted of rats with perirhinal cortex lesions (*n* = 17) and sham controls (*n* = 12). Likewise, cohort B consisted of rats with perirhinal cortex lesions (*n* = 18) and sham controls (*n* = 13). The surgical procedures were identical for both cohorts. All rats were anaesthetized throughout the surgery with isofluorane (5% for induction, and 2% thereafter). The rats were placed in a stereotaxic frame (David Kopf Instruments, Tujunga, CA, USA), with the incisor bar set at +5.0 mm to the horizontal plane. A sagittal incision was made in the scalp, and the skin was retracted to expose the skull. A dorsal craniotomy was made directly above the target region, and the dura was cut to expose the cortex.

The perirhinal cortex lesions were made by injecting a solution of 0.09 m *N*‐methyl‐d‐aspartate (Sigma, Poole, UK) dissolved in phosphate‐buffered saline (pH 7.4) in three sites in both hemispheres with a 26 gauge, 1‐μL Hamilton syringe (Hamilton, Bonaduz, Switzerland). The volumes of *N*‐methyl‐d‐aspartate injected were 0.22 μL for the rostral injections and 0.20 μL for the middle and caudal injections. The injection coordinates (in mm) relative to bregma were: (i) AP −1.8, ML ±5.9, DV −9.3; (ii) AP −3.4, ML ±6.2, DV −9.5; and (iii) AP −5.0, ML ±6.3, DV −8.9. These injections were made at a rate of 0.10 μL/min, with the needle being left in place for a further 4 min after each injection. After every surgical procedure, the skin was sutured together over the skull, and antibiotic powder was applied to the wound (Dalacin; Sandwich, Pfizer, UK). All rats received 5 mL of glucose saline subcutaneously, and were then placed in a heated box until they showed signs of recovery. For analgesia, Metacam (Boehringer, Inbelheim, DR, UK) was injected on three postoperative days. The surgical control groups received identical treatment, except that the dura was repeatedly perforated with an empty Hamilton syringe, so that no fluid was infused into the brain.

Prior to resuming behavioural testing, all rats were given time to recover (at least 2 weeks) and regain their preoperative weight. When behavioural testing resumed, the rats were put on a restricted diet to ensure that their weights were maintained at 85% of their free‐feeding weight.

### Apparatus

For all behavioural tests, the apparatus was a bow‐tie‐shaped maze. For Experiments 1, 2, and 6, the apparatus was identical to that described in previous studies (Albasser *et al*., 2010, 2011a,b). In Experiments 3, 4, and 5, a modified version of the bow‐tie maze was used. The modifications were designed to aid the video recording of behaviour within the maze, while also reducing background interference (visual or auditory) that might distract the rats.

The bow‐tie maze used in Experiments 1, 2 and 6 (Albasser *et al*., 2010) had steel walls and a wooden floor (Fig. 1). The maze was 120 cm in length, 50 cm in width, and 50 cm in height. Each end of the apparatus formed a triangular arena, the apices of which were joined by a narrow corridor (width, 12 cm). An opaque sliding door, which could be opened by the experimenter, was set in the middle of the corridor. The far wall of each triangular arena contained two recessed food wells, 3.5 cm in diameter and 2 cm in depth. A short, opaque dividing wall that protruded 15 cm from the middle of the end wall separated the two food wells. This wall stopped rats running directly between the two food wells.

The modified apparatus (Experiments 3, 4, and 5) retained the same shape and dimensions as the original bow‐tie maze described by Albasser *et al*. (2010). The modified maze, which was made from medium‐density fibreboard, was painted matt grey. Two recessed food wells, 3.5 cm in diameter and 2 cm in depth, were located 20 cm from both end walls. Between the two food wells was a metal dividing wall that protruded 15 cm from the middle of the end wall. At each end of the maze, doors in the far walls allowed the experimenter to introduce/remove rats, objects and food rewards without having to lean above the apparatus. Another modification was the addition of an opaque white plastic ceiling with a circular opening (diameter, 5 cm) at each end of the maze. A camera protruded through each circular opening in the ceiling, so allowing the viewing and recording of the activity within the maze. Each half of the maze was lit by three LED lights (each 30 cm in length) placed in a triangular formation attached to the ceiling at each end of the maze. The roof of the maze was covered with a blackout cloth. Monitors allowed both ends of the maze to be viewed.

In Experiments 1–6, numerous junk objects were used, each differing in shape, texture, size, and colour. Every object was large enough to cover a food well, but light enough to be displaced. Any object with an obvious scent was excluded. Enough objects were used to ensure that no object was repeated across experiments. All objects had multiple, identical copies, so that different copies of the same object were always used when an object was repeated within a session. All objects were cleaned with alcohol wipes after each session.

### Behavioural protocols

#### Experiment 1 – Simultaneous novelty preference test, short retention delays (pre‐surgery)

##### Pre‐training

The rats (cohort A) were pre‐trained for 7 days so that they would run from one side of the bow‐tie maze to the other, and displace an object covering a food well in order to reach sucrose pellets (45 mg; Rodent Diet; Noyes, Lancaster, NH, USA). Pre‐training (Albasser *et al*., 2010; Olarte‐Sánchez *et al*., 2014) was complete when rats would quickly shuttle between the two ends of the maze (i.e. as soon as the central sliding door was opened) and would displace an object to reach sucrose pellets. Over the course of pre‐training, four different pairs of objects were used, e.g. a pair of plain, plastic Lego blocks. On each pre‐training trial, two identical objects were placed at the same end of the maze.

##### Novelty preference (short retention delays)

The single test session contained 10 test trials. Apart from Trial 0, the rat could freely explore two objects, one novel and the other familiar, for a total of 1 min on each trial (Figs 1 and 2). Every object covered a baited food well to ensure that all objects were initially approached. To start the session, a rat was placed on one side of the maze (Trial 0), where a food well contained a single sucrose pellet (45 mg) that was covered by a novel object (object A_1_). The other food well at that end of the maze also contained a single food reward, but was covered by an object that was familiar, as it had been used repeatedly during pre‐training (e.g. a Lego block). The rat remained in that end of the maze (with object A_1_) for 1 min. The central sliding door was then pulled open, and the rat ran to the opposite side of the maze to initiate Trial 1 (Figs 1 and 2).

In Trial 1, the rat had a free choice between the now familiar object A_2_ and novel object B_1_ (Fig. 2), each covering a food well containing a single sucrose pellet. Both objects were simultaneously available for the rat to explore for a total of 1 min. The central sliding door was then opened to reveal two more objects for exploration at the opposite end of the maze (the now familiar object B_2_ vs. novel object C_1_) (Trial 2; Fig. 2). Both objects covered a reward pellet. The placement of the novel object varied from left to right according to a pseudorandom schedule. The order of the particular objects used in the test was reversed for half of the rats. This counterbalancing ensured that the novel object in any given pair was reversed, so that, for half of the rats in the trial that paired together the following two objects, a toy and a can, the can was the novel object. For the remaining rats, the toy was the novel object. Duplicate objects were used throughout to avoid odour contamination (e.g. A_1_ and A_2_).

All of the remaining experiments took place after recovery from surgery.

#### Experiment 2 – Simultaneous novelty preference test, short retention delays (post‐surgery)

This experiment was an exact replication of Experiment 1, except with a different set of objects (Fig. 2). Once again, in each of the 10 trials, the rats (cohort A) could spontaneously explore a novel object and an object made familiar because the rat had experienced it on the preceding trial. Each object covered a food well containing a single sucrose pellet. Testing was resumed between 2 weeks and 3 weeks after surgery. All rats were first given 1 day of habituation to the maze to ensure they would still shuttle between the two ends of the maze and displace objects over the food wells.

#### Experiment 3 – Simultaneous novelty preference test, 1‐h retention delay

This experiment consisted of two distinct phases (Fig. 2). The procedure for the sample phase was identical to that in Experiments 1 and 2. Testing therefore began with Trial 0, in which each rat (cohort A) was introduced to a novel object (A_1_). This trial was followed by six trials, each lasting for 1 min, and consisting of one novel and one familiar object (e.g. Trial 1, A_2_ vs. B_1_; Fig. 2). At the end of the sample phase, the rats were placed back in their home cage and taken back to the holding room for 1 h.

After 1 h, the rats were brought back to the test room in their home cages. The retention test phase involved a further six trials. On each trial (7–12), a novel object was presented along with a ‘familiar’ object, which had been experienced during the previous sample phase (Trials 1–6) 1 h before (e.g. A_3_ vs. Z). The location of the novel objects varied from left to right. The objects were counterbalanced so that the objects used in the sample phase for half of the rats were used as novel objects in the test phase for the remaining rats.

A feature of Experiment 3 (and of Experiment 4) was that the exploration data in the test phase were analysed with ethovision (Noldus, Wageningen, The Netherlands). The intention was to formalize the scoring. For this reason, each object was placed on a mark set immediately behind each baited food well (rather than immediately over the food well). This placement meant that the rats did not move the objects.

#### Experiment 4 – Simultaneous and sequential tests of novelty preference, 1‐h retention delay

This experiment began with a sample phase, which was followed 1 h later by three different trial types that contrasted novelty preference in two ways (test phase). The first was to present simultaneously one novel and one familiar object, i.e. as in Experiments 1–3. The second (sequential) was to give pairs of identical objects that were either both novel or both familiar (Figs 1 and 2).

In the sample phase, the rats in cohort A underwent eight trials, each 1 min apart. Testing was identical to that in the first phase of Experiment 3, except that, in each trial, the rats received a pair of identical objects to explore (Fig. 2). Each trial involved a different pair of duplicate objects (Trial 1, A_1_ vs. A_2_; Trial 2, B_1_ vs. B_2_; and so on). The rats were then returned to their home cage, and taken back to the holding room for 1 h. As in Experiment 3, the objects were placed immediately behind the baited food well.

The test phase contained three different trial types (Fig. 2), presented in a counterbalanced order. Four trials involved a single object from the sample phase paired with a novel object (e.g. B_3_ vs. J), so giving a simultaneous recognition test. Four more trials involved a pair of identical objects that were new to the rat (e.g. K_1_ vs. K_2_), i.e. novel pairs. A further four trials involved pairs of identical objects that were the same as the pairs used in the sample phase (e.g. A_3_ vs. A_4_), i.e. familiar pairs. As in Experiments 1–3, each trial lasted for 1 min. The use of multiple copies of the same object (e.g. A_1_, A_2_, A_3_, and A_4_) avoided odour contamination.

The placement of the objects in the two phases and whether they were novel or familiar in the test phase were counterbalanced. This involved three complementary sequences that exchanged objects across phases and test types. The food well in front of each object was baited on every trial. Each rat was tested only once.

#### Experiment 5 – Habituation to repeated presentations of object pairs

This experiment examined exploration levels for novel objects, followed by repeat presentations of the same objects to determine whether rats with perirhinal cortex lesions show the expected reduction in exploration with stimulus recognition.

All rats (cohort A) underwent 16 trials in the modified bow‐tie maze in one continuous session (Fig. 2). In Trial 1, the rat could explore two dissimilar novel objects (A_1_ and B_1_) for up to 1 min (both objects were placed behind a food reward). After 60 s, the central sliding door was opened, and the rat ran to the opposite end of the maze, which contained duplicates of the same objects (A_2_ and B_2_). The next trial, after 60 s, again involved the same familiar pair of objects (A_3_ and B_3_). The procedure was repeated for a fourth trial (A_4_ and B_4_). In the fifth trial, both objects were replaced, so the rat was now confronted with two novel objects (C_1_ and D_1_). These same objects (C and D) were repeated over the next three trials. Over the 16 trials, each rat received four sets of four trials, each set consisting of repeats of the same object pair. The side positions of the objects changed between trials. Each object was placed over a baited food well.

#### Experiment 6 – Paired presentation of novel objects

This experiment again sought to assess the sensitivity of rats with perirhinal cortex lesions to novel objects that did not need to be discriminated. Consequently, the rats (cohort B) underwent 20 consecutive trials in the original bow‐tie maze, in which each trial contained two different objects placed over the food wells, but now every object was novel for the rat (Fig. 2). This procedure created the opportunity for the rats to explore the maximum number of novel objects (40) over the 20‐trial session.

### Behavioural analyses (Experiments 1–6)

Animals were video‐recorded throughout training. Object exploration was defined as directing the nose at a distance < 1 cm from the object, with the vibrissae moving, and/or touching it with the nose or the paws. Object exploration was not scored when rats sat on the object, when rats used the object to rear upwards with the nose of the rat facing the ceiling, or when rats chewed the object. With the exception of Experiments 3 and 4 (test phases), the duration of object exploration was determined by holding down a keypad on a computer. For Experiments 3 and 4, analyses with ethovision provided the behavioural data from the test phase. For these same two experiments, in addition to the total duration of exploration (Table 1), individual, discrete bouts of exploration were also recorded (Ennaceur *et al*., 2009). A bout was defined as the initiation of exploratory behaviour. The goal was to provide a more complete picture of behavioural discrimination (Silvers *et al*., 2007; Bernice & Raber, 2008) for those experiments that most clearly contrasted the different testing protocols. The results from these additional measures are summarized in Table 2 but not included in Results.

**Table 1:** Comparisons of total object exploration times by the two groups [perirhinal cortex lesion (PRh) and sham surgery] in Experiments 2–6

	Sham vs. PRh (cohort A)
(2) N and F	NS *P* > 0.1
(3) N and F sample phase	NS *P* > 0.1
(3) N and F test phase	NS *P* > 0.1
(4) N^a^ and N^a^ sample phase	NS *P* > 0.05*
(4) N^a^ and N^a^ sequential test phase	NS *P* > 0.1
(4) F^a^ and F^a^ sequential test phase	NS *P* > 0.1
(4) N and F simultaneous test phase	NS *P* > 0.05*
(5) N^x^ and N^y^	NS *P *>* *0.1
	Sham vs. PRh (cohort B)
(6) N^x^ and N^y^	NS *P* > 0.1

NS, no significant group difference (i.e. *P* > 0.05). The various test conditions comprised: novel with familiar objects (N and F), novel with novel objects (N and N), and familiar with familiar objects (F and F). The designation N^a^ and N^a^ represents two identical novel objects, the designation F^a^ and F^a^ represents two identical familiar objects, and the designation N^x^ and N^y^ represents two different novel objects. Note, that in the two cases where the group difference was 0.1 > *P* > 0.05 (asterisked), it was the PRh group that showed the higher exploration levels.

**Table 2:** Summary of findings from the test phase (1‐h retention) of Experiments 3 and 4 (cohort A)

	Sham	PRh
Experiment 3		
Group difference D1, D2	* ^,^ **	
Above chance D1, D2	** ^,^ **	* ^,^ *
Time N vs. F†	**	NS
Bout numbers N vs. F	NS	NS
Bout duration N vs. F	**	NS
Total bouts		↑
Experiment 4 simultaneous		
Group difference D1, D2	** ^,^ **	
Above chance D1, D2	** ^,^ **	NS*
Time N vs. F†	**	NS
Bout numbers N vs. F	NS	NS
Bout duration N vs. F	**	NS
Total bouts		↑
Experiment 4 sequential		
Group difference D1, D2	NS, NS	
Above chance D1, D2	** ^,^ **	** ^,^ **
Time N vs. F.†	**	**
Bout numbers N vs. F	**	**
Bout duration N vs. F	NS	*
Total bouts		↑

F, familiar object; N, novel object; PRh, perirhinal cortex lesion group; ↑, significant (*P* < 0.05) increase in frequency as compared with the other group. ‘Above chance’ refers to whether each group of rats discriminated the novel objects, i.e. that a one‐sample *t*‐test (D1, D2) is above the chance score of zero. **P < 0.05; *0.05 < *P* < 0.1; NS, *P* > 0.1. ^†^Those time comparisons for which the data may not be independent, and so are not detailed in Results.

For tests of spontaneous object preference, two performance indices are often calculated: D1 and D2 (Ennaceur & Delacour, 1988). D1 is the difference between the exploration time devoted to the novel object and that devoted to the familiar object (novel minus familiar). The second measure (D2) uses the same difference in exploration times (i.e. D1), but then divides D1 by the total duration of exploration given to both the novel and familiar objects. In the present study, the D2 scores were only calculated at the end of each session or at the end of a block of trials (‘updated D2’). Consequently, the differences in exploration times for the novel and familiar objects were summed across consecutive trials and then divided by the total amount of exploration for the same series of trials. As D2 compensates for individual changes in total amounts of exploration, the Results section focuses on this index, although, in practice, the findings for D1 and D2 mirrored each other very closely (Table 2). The D2 ratio can vary between +1 and −1, with a positive ratio showing a preference for novel objects, and a ratio of 0 corresponding to no preference, i.e. chance. Throughout the study, all behavioural scoring was blind; that is, the experimenter did not know the group allocation of individual rats.

### Statistical analyses (Experiments 1–6)

Those group comparisons based on Student *t*‐tests between recognition index scores (D1 and D2) were one‐tailed, reflecting the expectation that control rats would outperform rats with perirhinal cortex lesions. In contrast, group comparisons of total exploration times were two‐tailed. To examine whether the rats could solve the recognition memory tasks, one‐sample *t*‐tests were used to determine whether group D2 scores were above zero, i.e. chance (one‐tailed, to reflect the question being tested).

For the sequential tests, a mixed anova was used to compare exploration times for novel and familiar objects across the two groups. On one occasion, simple effects were reported when there was a main effect but no interaction. This analysis concerned a specific question under investigation (Howell, 1982, p. 326). It should be noted that the exploration times for simultaneous choice tasks are not, strictly speaking, independent, as a rat cannot be with both objects at the same time. (Normally, rats spent < 10 s of each 60‐s trial exploring, so this may be a minor concern.) Nevertheless, time spent with novel objects was not compared directly with the time spent with simultaneously presented familiar objects. Instead, for the simultaneous discrimination tasks, separate group comparisons were made for the time spent with novel objects and for the time spent with familiar objects. The alpha level was set throughout at *P* ≤ 0.05.

## Results

**Figure 3:**
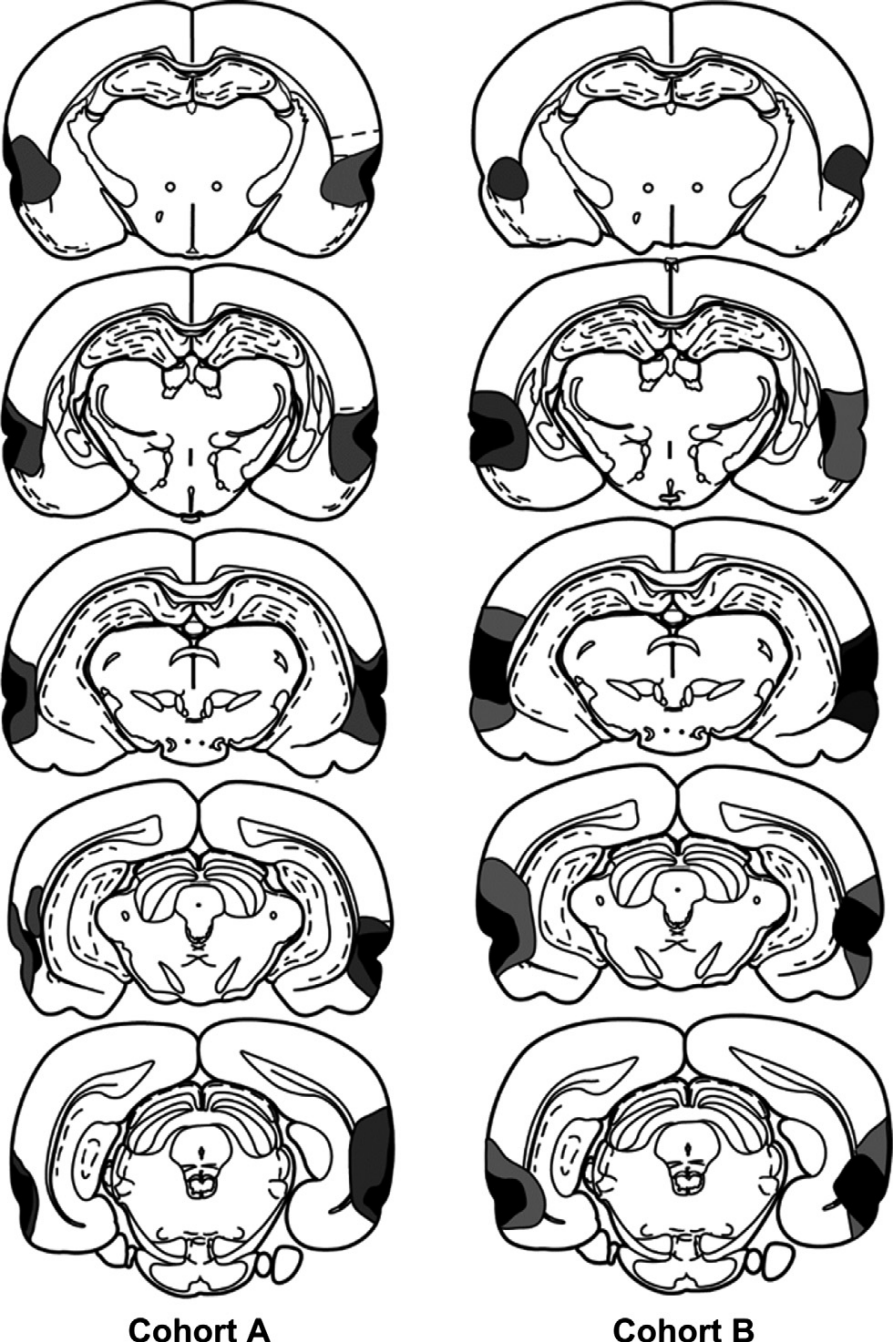
Diagrammatic reconstructions of the perirhinal cortex lesions, showing the individual cases with the largest (grey) and smallest (black) lesions in cohort A and cohort B. The most rostral coronal section is at the top. The sections are ~1 mm apart in the AP plane.

The perirhinal cortex lesions in cohort A were centred in the rhinal sulcus, causing extensive bilateral damage to areas 35 and 36 (Fig. 3). Of the 17 rats receiving perirhinal cortex lesions, one was rejected because of excessive bilateral tissue sparing. Figure 3 depicts the two individuals from the remaining 16 cases with the largest and smallest extent of perirhinal cortex damage. The lesions typically involved almost the full anterior–posterior extent of areas 35 and 36 (Burwell, 2001). The mean percentage of perirhinal cortex loss was 76.0% (range 53.7–95.0%). A frequent feature was the addition of some cell loss in the most dorsal parts of the piriform cortex and lateral entorhinal cortex, i.e. those parts adjacent to area 35. Of the 16 rats, seven had some unilateral damage to that part of CA1 immediately adjacent to the fundus of the rhinal sulcus. In a further seven cases, there was bilateral damage in this location. The CA1 cell loss was typically very restricted, being located next to the more caudal rhinal sulcus, and often only visible on a couple of sections. The final group numbers in cohort A were as follows: perirhinal cortex lesion, 16; and sham, 12.

The perirhinal cortex lesions in cohort B were similar to those in the previous cohort (Fig. 3). Consequently, there was often almost complete cell loss along the full length of the perirhinal cortex. Seven of the 18 cases were excluded, however, as they had appreciable bilateral damage in the hippocampus (CA1 field) in addition to the targeted perirhinal cortex. The mean percentage of total perirhinal cortex loss in the remaining 11 rats was 84.4% (range 70.1–98.3%). Of these 11 rats, five had only unilateral cell loss in the CA1 field immediately adjacent to the caudal part of the rhinal sulcus, and the remainder had extremely restricted CA1 cell loss (typically on just one or two sections) in the other hemisphere. In these same 11 cases, there was consistent cell loss in those parts of the piriform and lateral entorhinal cortices immediately adjacent to the perirhinal cortex. In four of these cases, there was unilateral damage in the most superior part of the lateral amygdala nucleus, and that part of Te2 closest to area 36 was also often partly damaged. One sham rat was excluded because it had unilateral damage to the rostral half of the dorsolateral cortex for, unknown reasons. The final group numbers for cohort B were as follows: perirhinal cortex lesion, 11; and sham, 12.

### Experiments 1 and 2 – Simultaneous novelty preference test, short retention delays (cohort A)

Pre‐surgical comparisons (Experiment 1) between the rats subsequently constituting the sham and perirhinal cortex lesion groups showed no group difference in their D2 scores (both *t* < 1). Likewise, there was no group difference in their total object exploration times (familiar plus novel) (*t* < 1; shams to be, mean of 147.1 s; perirhinal cortex lesions to be, mean of 143.5 s).

Following surgery (Experiment 2), the D2 index scores did not distinguish (*t* < 1) the sham group from the perirhinal cortex lesion group at the short retention delays (between 0 s and 60 s). Both groups successfully identified the novel objects (one‐sample *t*‐test: both groups *P* < 0.001). Although the perirhinal cortex lesion rats tended to explore more than the sham rats (novel plus familiar objects, mean: perirhinal cortex lesion, 113.9 s; sham, 96.7 s), this difference was not significant (*t*_26_ = 1.65, *P* = 0.11).

### Experiment 3 – Simultaneous novelty preference test, 1‐h retention delay (cohort A)

**Figure 4:**
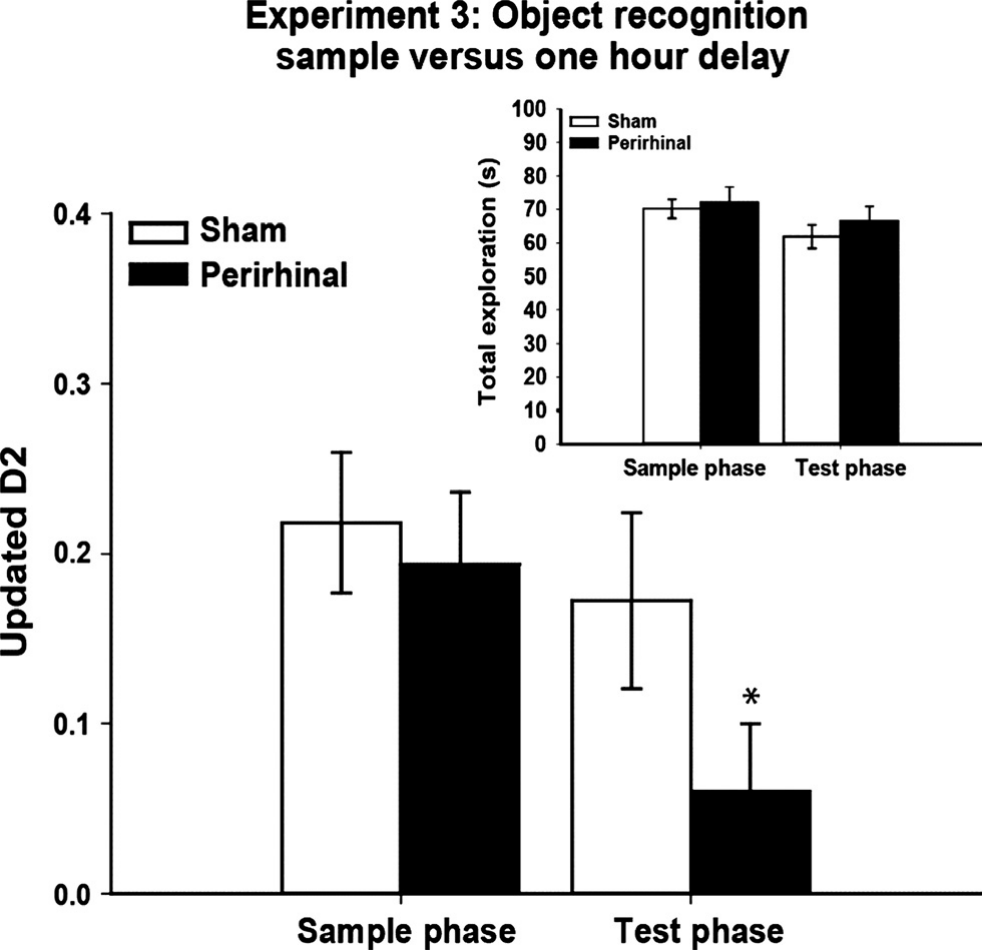
Experiment 3: sample phase and test phase (1‐h delay) performance on a simultaneous test of object recognition by rats with perirhinal cortex lesions (black) and their controls (white). The main graph depicts novelty preference (Updated D2) in both the sample phase (retention delay of <60 s) and the test phase (retention delay of 1 h). One novel and one familiar object were simultaneously presented on each trial (forced‐choice). The inset shows total object exploration levels during the sample and test phases. The data shown are mean ± standard error of the mean. *P ≤ 0.05 for group difference in the test phase.

The perirhinal cortex lesions impaired performance in the test phase, i.e. after a 1‐h retention (Fig. 4). Consequently, the D2 scores of this group were lower than those of the sham group (D2, *t*_26_ = 1.75, *P* = 0.046). Whereas the sham rats distinguished the novel object (D2, one‐sample *t*‐test, *t*_11_ = 3.33, *P* = 0.0035), the corresponding preference scores of the perirhinal cortex lesion rats narrowly failed to exceed chance (D2, *t*_15_ = 1.53, *P* = 0.074). The cumulative exploration times (novel plus familiar) during the sample phase (mean: sham, 70.2 s; perirhinal cortex lesion, 72.2 s) and the test phase (sham, 61.9 s; perirhinal cortex lesion, 66.5 s) did not separate the two groups (both *t* < 1). Table 2 shows the corresponding results obtained from examining bout numbers.

### Experiment 4 – Simultaneous and sequential tests of novelty preference, 1‐h retention delay (cohort A)

In phase 1 (sample phase), rats were given duplicate pairs of novel objects to explore (Fig. 2). The total exploration times of the perirhinal cortex lesion rats (95.7 s) appeared to be higher than those of the sham rats (79.0 s), although this difference was not significant (*t*_26_ = 1.85, *P* = 0.076).

In phase 2 (test phase), rats underwent trials that consisted of either: one novel and one familiar object (simultaneous discrimination), two novel objects (sequential), or two familiar objects (sequential). For the simultaneous trials, the exploration times for the novel and familiar stimuli within the same trials were compared separately. For the sequential trials, the total exploration times for the novel pairs and the familiar pairs were first determined and then compared. Table 2 shows the corresponding results obtained from examining bout numbers.

#### Simultaneous trials

**Figure 5:**
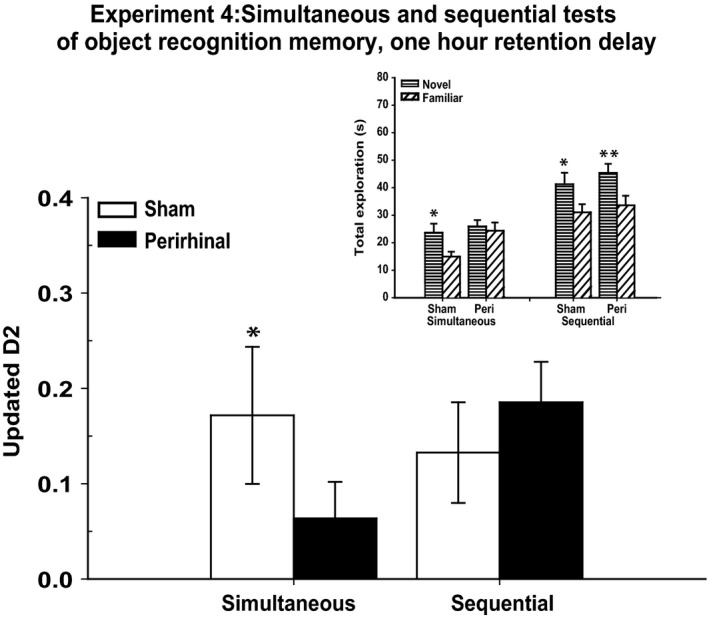
Experiment 4: simultaneous and sequential tests of object recognition, both with retention delays of 1 h. The main graph depicts novelty preference (Updated D2) when testing was performed with simultaneous (forced‐choice) and sequential (yes/no) protocols in the same session. Asterisks indicate group difference in the test phase (*P ≤ 0.05). The inset shows total times for the novel and familiar objects in the test phase for both simultaneous and sequential protocols. Asterisks indicate the significance of the within‐subject comparisons for time spent with novel objects and time spent with familiar objects (*P ≤ 0.05, **P ≤ 0.01). The data shown are mean ± standard error.

Following the 1‐h retention period, the perirhinal cortex lesion rats showed novelty preference deficits (Fig. 5). The D2 index was significantly lower in the perirhinal cortex lesion group than the sham group (D2, *t*_26_ = 1.96, *P* = 0.031). Whereas the sham rats discriminated the novel from the familiar objects (one‐sample *t*‐test, D2, *t*_11_ = 2.74, *P* = 0.0095), the performance of the perirhinal cortex lesion rats failed to exceed chance (D2, *t*_15_ = 1.50, *P* = 0.083). Although the perirhinal cortex lesion rats appeared to show higher overall levels of object exploration than the sham rats, this difference was not significant (*t*_26_ = 1.77, *P* = 0.089). The two groups did not differ in the time spent exploring the novel objects (Fig. 5; *t* < 1). In contrast, the perirhinal cortex lesion rats spent more time than the sham rats with the familiar objects (*t*_26_ = 2.58, *P* = 0.016, two‐tailed).

#### Sequential trials

Both groups discriminated the novel from the familiar objects according to the D2 index (sham, *t*_11_ = 2.22, *P* = 0.024; perirhinal cortex lesion, *t*_15_ = 3.74, *P* = 0.001; Fig. 5). There was no group difference for D2 (*t* < 1). Overall, exploration times were higher for the novel objects than for the familiar objects (*F*_1,26_ = 15.20, *P* = 0.001), but there was no group difference (*F* < 1) or interaction (*F* < 1) on this measure (Fig. 5), with both groups spending more time with the novel pairs of objects (maximum *P* = 0.017).

To compare the sensitivity of the two test methods, the D2 scores of the sham group were compared for the simultaneous and sequential tests. There was no evidence that these scores differed (*t* < 1).

### Experiment 5 – Habituation to repeated presentations of object pairs (cohort A)

**Figure 6:**
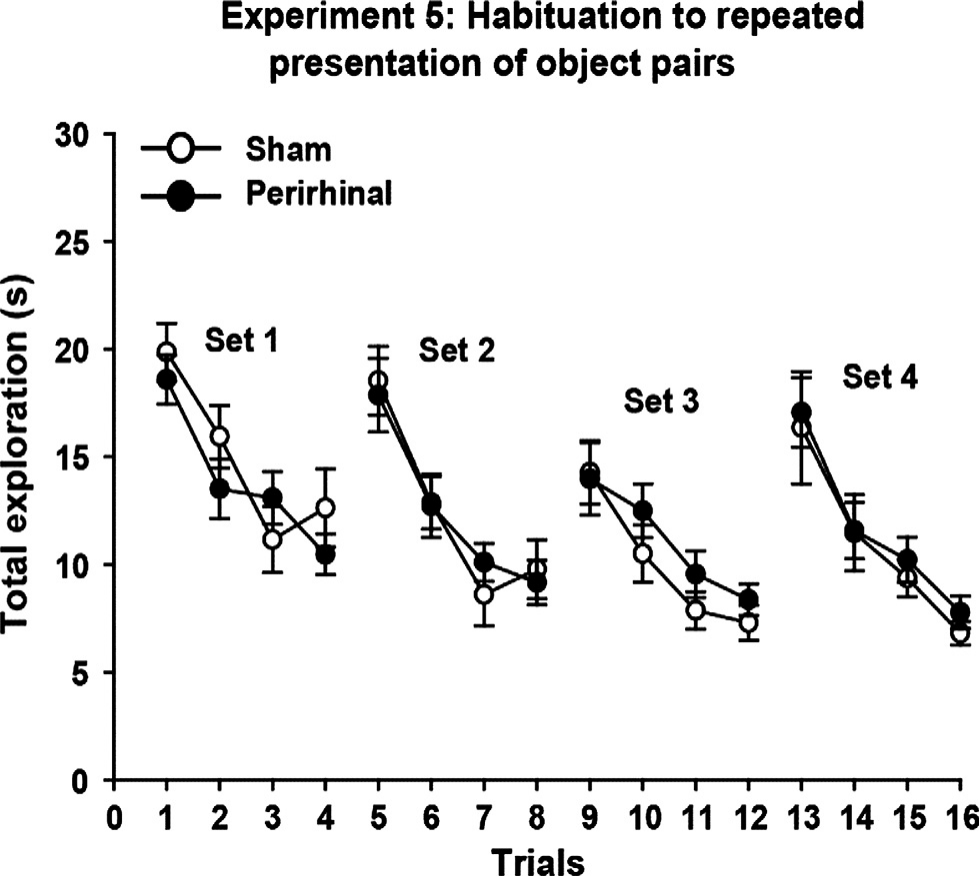
Experiment 5: habituation to repeated presentation of object pairs. The graphs show the duration of object exploration for a pair of novel objects (Trials 1, 5, 9, and 13) followed by three repeats of those same objects (Sets 1–4). The data shown are mean ± standard error of the mean.

Both groups of rats decreased their levels of object exploration as pairs of objects were repeated, but showed a marked increase with the introduction of new object pairs (Fig. 6). The levels and patterns of performance of the sham and perirhinal cortex lesion groups seemed almost indistinguishable. Consequently, there was no overall group difference in exploration levels (*F* < 1), which contrasted with a marked effect within each set of trials (*F*_3,78_ = 47.8, *P* < 0.001), as repetition of the same object led to lower exploration levels (Fig. 6). There were no interactions between surgical group and trial number (1–4) or set number (all *F* < 1).

### Experiment 6 – Paired presentation of novel objects (cohort B)

The exploration data were divided between the first 10 and second 10 pairs of dissimilar novel objects. The overall time spent exploring (Trials 1–20) appeared to be higher in the perirhinal cortex lesion rats (means: perirhinal cortex lesion, 195.8 s; sham, 162.3 s), but there was no significant group difference (*F*_1,21_ = 2.76, *P* = 0.11). Likewise, there was no effect of trial block (*F*_1,21_ = 1.95, *P* = 0.18) and no interaction between these factors (*F*_1,21_ = 2.15, *P* = 0.16).

## Discussion

Perirhinal cortex lesions in rats are presumed to impair recognition memory, as demonstrated by deficits on delayed non‐matching‐to‐sample problems (Mumby & Pinel, 1994), and the difficulty observed in discriminating novel from familiar objects in spontaneous preference tests (Brown, 1996; Ennaceur *et al*., 1996; Winters *et al*., 2008; Warburton & Brown, 2010; Kinnavane *et al*., 2015). Prior to a spontaneous preference test, rats must first be familiarized with one of the objects (sample phase), which is subsequently paired with a novel object in the test phase. It is therefore remarkable that, in those same studies showing apparent recognition deficits, rats with perirhinal cortex lesions often show normal levels of novel object exploration during the sample phase (e.g. Ennaceur *et al*., 1996; Aggleton *et al*., 1997; Moran & Dalrymple‐Alford, 2003; Winters *et al*., 2004; Barker *et al*., 2007; Bartko *et al*., 2007a,b; Mumby *et al*., 2007; Albasser *et al*., 2009, 2015; McTighe *et al*., 2010). Two different explanations have been given for why perirhinal cortex lesions can spare this example of novelty detection, but impair the subsequent test phase, when rats are selecting between one novel and one familiar object.

One explanation is that rodents with perirhinal cortex lesions still detect novelty or familiarity, but are deficient at discriminating which specific stimulus is novel or familiar (Albasser *et al*., 2015). As only novel stimuli are present in the sample phase, this explanation predicts that exploration levels will appear normal. Recognition deficits then appear in the test phase, when novel and familiar objects have to be discriminated. A second explanation is that rodents with perirhinal cortex lesions are highly sensitive to visual interference (Bussey *et al*., 2005; Cowell *et al*., 2010; McTighe *et al*., 2010). This interference account assumes that the restricted home cage conditions prior to testing ensure low interference, so protecting sample phase performance, despite the perirhinal cortex lesion (McTighe *et al*., 2010; Romberg *et al*., 2012). Consequently, the object in the initial sample trial will be correctly detected as novel, generating appropriate exploration levels. This explanation further assumes that objects encountered on this first sample trial are sufficient to produce marked interference effects on the following test trial, so causing subsequent recognition deficits (Romberg *et al*., 2012).

Predictions from the two explanations (discrimination or interference) were compared in two ways. One approach was based on exploration levels, and the other was based on comparing the outcome of simultaneous test procedures with that of sequential test procedures.

### Object exploration levels after perirhinal cortex lesions

The discrimination account predicts that overall exploration levels in novelty preference tests will remain close to normal, prompted by the presence of a novel object, even though the rat may struggle to isolate the particular novel stimulus (Albasser *et al*., 2015). In contrast, the interference account predicts that multiple, continuous trials should generate high levels of proactive interference, causing novel objects to appear familiar in rats with perirhinal cortex lesions (Cowell *et al*., 2010; McTighe *et al*., 2010). Consequently, exploration levels for novel objects will become abnormally low as testing progresses.

In contrast to what is predicted by the interference explanation, perirhinal cortex lesions did not significantly reduce overall object exploration in any of the nine training conditions (Table 1). In each of these conditions, the rats received multiple trials within a session, which presumably increased proactive interference. Across the various experiments, rats explored different combinations of objects: novel with familiar, familiar with familiar, and novel with novel (Table 1). For the last combination, there were two variants. In one variant, the rats explored two identical novel objects (Experiment 4, sample phase); in the other, the rats explored two different objects, both novel (Experiments 5 and 6). These near‐normal levels of object exploration are similar to the findings from a previous study that also used the bow‐tie maze (Albasser *et al*., 2015). In that study, data from the recognition test trials (novel vs. familiar) of four cohorts of animals with perirhinal cortex lesions were combined (giving *n* > 40) to increase statistical power. The overall finding was a very modest decrease in object exploration, despite multiple trials creating conditions for raised interference (Albasser *et al*., 2015). It was this modest decrease that prompted the inclusion of cohort B (Experiment 6), which helped to show how rats with perirhinal cortex lesions can show seemingly normal levels of exploration for novel objects when they are not in direct competition with familiar objects.

In Experiment 5, not only did rats with perirhinal cortex lesions show normal exploration levels for novel objects, but the repetition of these same objects over successive trials also led to comparable levels of habituation. This pattern was followed by a return to higher exploration levels when the now familiar objects were replaced by two novel stimuli (Fig. 6). Although only short inter‐trial intervals were used, other studies using both short (Robinson *et al*., 2009; Albasser *et al*., 2011a, 2015) and longer (Mumby *et al*., 2007) inter‐trial intervals have similarly found that rats with perirhinal cortex lesions show normal levels of exploration in response to a novel stimulus, which then decline at control rates when that same stimulus is repeated. This sparing has been found in rats that are impaired on simultaneous object recognition at even the shortest delays (Albasser *et al*., 2011a). One exception may be a failure to detect the novelty of tastes following perirhinal cortex lesions (Ramos, 2015).

A feature of Experiments 3 and 4 was the inclusion of other aspects of exploratory behaviour (Silvers *et al*., 2007; Ennaceur *et al*., 2009). Analysis of the sham rats indicated that bout duration (longer for novel objects; Experiment 3) and bout numbers (more for novel objects; Experiment 4) could help to distinguish novel from familiar stimuli, but these measures were not consistent predictors of object novelty (Table 2). An unexpected finding was that perirhinal cortex lesions sometimes increased the total number of exploration bouts. This increase occurred with both simultaneous and sequential testing (Table 2). The cause of this behavioural change is uncertain, especially as perirhinal cortex lesions rarely cause hyperactivity (but see Wiig & Bilkey, 1994). One explanation may reflect the confusion of the rats regarding the identity of novel objects, leading to greater switching behaviour. Irrespective of the explanation, the results suggest that the impact of perirhinal cortex lesions on exploratory behaviour is multi‐levelled (Ennaceur *et al*., 2009), so reinforcing the value of complementary measures, such as delayed non‐matching to sample (Mumby & Pinel, 1994; Steckler *et al*., 1998; Kinnavane *et al*., 2015), to help assess the impact of perirhinal cortex lesions on recognition memory.

### Simultaneous vs. sequential modes of object preference testing

The second method for comparing the two explanations (discrimination or interference) involved testing both simultaneous (forced‐choice) and sequential (yes/no) modes of object recognition. The discrimination account predicts that abnormal behaviour should be most evident when a novel and a familiar object are both present in the same trial, i.e. simultaneous testing. In contrast, the interference hypothesis predicts that both simultaneous and sequential testing should be sensitive to perirhinal cortex damage. The present findings provided a clear distinction. The rats with perirhinal cortex lesions were impaired on simultaneous object recognition when there was a 1‐h delay between sample and test (Experiments 3 and 4). In contrast, the same lesioned rats could still discriminate novel from familiar objects, not differing from the controls, when subjected to sequential trials after a 1‐h retention delay. The spared ability of rats with perirhinal cortex lesions to discriminate novel from familiar stimuli when they are presented in separate (sequential) trials extended to bout numbers (more for novel stimuli, both groups) and to the mean duration of each bout (longer for novel stimuli) (Table 2).

The apparent sparing of recognition memory when object recognition is tested sequentially (Experiment 4) could potentially result from this testing procedure being easier for the rats than simultaneous testing, an effect that could be exacerbated if the perirhinal cortex lesions are insufficient. In fact, neither the D1 scores nor the D2 scores of the sham group were significantly lower for simultaneous than for sequential tests in Experiment 4, indicating that task difficulty does not explain the different pattern of results. With respect to lesion size, previous cohorts with comparably sized perirhinal cortex lesions have been shown to be consistently impaired at simultaneous object recognition, sometimes at the shortest retention delays (see Aggleton *et al*., 2010; Albasser *et al*., 2010, 2011b, 2015). Other cohorts, such as those in the present study, appear to be more impaired after longer retention delays (Ennaceur *et al*., 1996; Norman & Eacott, 2004; but Bartko *et al*., 2007a).

Although the present findings help to explain the numerous studies that have reported normal levels of object exploration in the sample phase by rats with perirhinal cortex lesions, they contrast with the finding (McTighe *et al*., 2010) that rats with perirhinal cortex lesions will treat novel objects as if they are familiar. Despite these contrasting outcomes, the retention interval for recognition testing in that study (McTighe *et al*., 2010) was 1 h, i.e. the same as in the present study. Furthermore, this same interval was sufficient to impair the rats with perirhinal cortex lesions on forced‐choice object preference (Experiments 3 and 4). In the study by McTighe *et al*. (2010), reducing interference between sample and test (by placing the rats in a dark, quiet environment) restored normal exploration levels for pairs of novel objects in the rats with perirhinal cortex lesions. However, in the present study, multiple sample trials were used (Experiment 4; Fig. 2) and the retention period remained in the light. Consequently, it is most unlikely that the spared performance on sequential trials in the present study reflected a lack of interference between first encountering a sample object and its subsequent test. Although the surgical procedures used in the two studies appeared to be very similar, creating perirhinal cortex lesions of comparable extent, additional lesions were made by McTighe *et al*. (2010), involving the postrhinal cortex. This surgical difference leaves the possibility that postrhinal tissue loss, which affects memory for context (Bucci *et al*., 2000; Norman & Eacott, 2005), influenced exploratory behaviour.

### Summary and conclusion

Experiments 1–6 compared object novelty judgements when varying demands were made on the discrimination of individual objects. Like the comparison between object‐in‐place and object location tasks (Bussey *et al*., 2000; Warburton & Brown, 2010), where only the former is sensitive to perirhinal lesions, the results point to a pattern of deficits that occur when specific object identity is demanded (Bartko *et al*., 2007a,b; Diana *et al*., 2007; Ramos, 2014; Hales *et al*., 2015). Such findings could be incorporated into the ‘representational hierarchical model’ (Bussey *et al*., 2005), which assumes that the perirhinal cortex holds object‐level representations of complex stimuli, so helping to distinguish stimuli with overlapping features (Bussey & Saksida, 2007; Murray *et al*., 2007; Cowell *et al*., 2010; Ahn & Lee, 2015). Consequently, perirhinal cortex lesions force the use of simpler feature‐based representations, sometimes leading to raised proactive interference, as these representations contain more common elements.

The present findings provide new insights into the recognition deficit seen in rats with perirhinal cortex lesions (Brown & Aggleton, 2001). The first is that an accurate sense of novelty or familiarity is retained, despite the loss of more refined object‐level representations, along with their associated novelty/familiarity information. This conclusion has a possible parallel in human priming experiments, in which non‐declarative (implicit) signals of novelty, e.g. perceptual fluency, only guide recognition memory under limited conditions (Hamann & Squire, 1997; Verfaellie & Cermak, 1999; Voss *et al*., 2008; Voss & Paller, 2009). In the rat brain, candidate sites providing a less localized novelty signal include area Te2, the postrhinal cortex, and the entorhinal cortex (Zhu *et al*., 1995; Furtak *et al*., 2007; Albasser *et al*., 2011a; Ho *et al*., 2011; Kinnavane *et al*., 2014). The implication is that such novelty/familiarity information can be derived from multiple cortical sites, although it lacks sufficient specificity to guide the effective forced‐choice discrimination of objects. These same findings highlight the difficulty in equating the strength of a novelty preference with the level of recognition memory (Gaskin *et al*., 2010), as the outcome may be highly sensitive to procedural factors. The second insight is that, despite prior evidence for raised sensitivity to visual interference following perirhinal cortex lesions (Gilbert & Kesner, 2003; Bartko *et al*., 2010; McTighe *et al*., 2010; Albasser *et al*., 2015), there was no evidence that these lesions result in a default bias for novel stimuli to appear familiar.
